# Cytoplasmic Polyadenylation Element-Binding Protein 1 Post-transcriptionally Regulates Fragile X Mental Retardation 1 Expression Through 3′ Untranslated Region in Central Nervous System Neurons

**DOI:** 10.3389/fncel.2022.869398

**Published:** 2022-04-15

**Authors:** Souichi Oe, Shinichi Hayashi, Susumu Tanaka, Taro Koike, Yukie Hirahara, Ryohei Seki-Omura, Rio Kakizaki, Sumika Sakamoto, Yosuke Nakano, Yasuko Noda, Hisao Yamada, Masaaki Kitada

**Affiliations:** ^1^Department of Anatomy, Kansai Medical University, Hirakata, Japan; ^2^Department of Anatomy, Bio-Imaging and Neuro-Cell Science, Jichi Medical University, Shimotsuke, Japan; ^3^Biwako Professional University of Rehabilitation, Higashiomi, Japan

**Keywords:** cytoplasmic polyadenylation-binding protein 1, fragile X mental retardation 1, heat shock protein family A member 9, mitochondria, post-transcriptional regulation

## Abstract

Fragile X syndrome (FXS) is an inherited intellectual disability caused by a deficiency in Fragile X mental retardation 1 (*Fmr1*) gene expression. Recent studies have proposed the importance of cytoplasmic polyadenylation element-binding protein 1 (CPEB1) in FXS pathology; however, the molecular interaction between *Fmr1* mRNA and CPEB1 has not been fully investigated. Here, we revealed that CPEB1 co-localized and interacted with *Fmr1* mRNA in hippocampal and cerebellar neurons and culture cells. Furthermore, CPEB1 knockdown upregulated *Fmr1* mRNA and protein levels and caused aberrant localization of Fragile X mental retardation protein in neurons. In an FXS cell model, CPEB1 knockdown upregulated the mRNA levels of several mitochondria-related genes and rescued the intracellular heat shock protein family A member 9 distribution. These findings suggest that CPEB1 post-transcriptionally regulated *Fmr1* expression through the 3′ untranslated region, and that CPEB1 knockdown might affect mitochondrial function.

## Introduction

Fragile X syndrome (FXS) is the most common form of inherited intellectual disability that manifests with clinical features, including anxiety, autism symptoms, and poor attention, and physical features, including prominent ears, a long face, and hyperextensible finger joints (Hagerman et al., 2017; Niu et al., 2017; Bagni and Zukin, 2019; Okazaki et al., 2021). FXS is caused by a reduction in fragile X mental retardation protein (FMRP) expression levels. In particular, 200 CGG triplet repeats in *Fmr1* promoter region leads to enhanced DNA methylation and transcriptional inactivation (Saldarriaga et al., 2014; Hagerman and Hagerman, 2016). In the brain, FMRP is expressed in neuronal and glial cells and exerts multiple functions, including translation regulation, mRNA stability control and transport, and transcription modulation (Estes et al., 2008; De Rubeis and Bagni, 2010; Richter et al., 2015; Richter and Zhao, 2021). Therefore, loss of FMRP results in aberrant expression of numerous genes and causes developmental abnormalities during neurogenesis, dendritic arborization, and neuronal circuit formation (Guo et al., 2011; Aloisi et al., 2017; Goel et al., 2018).

To date, several studies have demonstrated that pharmacological approaches have the potential to ameliorate FXS pathology in *Fmr1* knockout mice, a commonly used FXS experimental model (Gantois et al., 2017; Dy et al., 2018). For example, metformin, an antidiabetic agent used in the treatment of type 2 diabetes mellitus, ameliorates core phenotypes, including social deficits, abnormal spine morphology, and exaggerated long-term depression of synaptic transmission, in *Fmr1* knockout mice (Gantois et al., 2017). Metformin mediates the amelioration by reducing pathologically hyperactivated mitogen-activated protein kinase 1 signaling to the normal level, and phosphorylating eukaryotic translation initiation factor 4E in *Fmr1* knockout mice (Gantois et al., 2017). Furthermore, a clinical study has revealed that metformin improved irritability, social avoidance, and aggression in FXS patients (Dy et al., 2018). In addition, Udagawa et al. (2013) showed that the altered phenotypes, such as translational homeostasis, dendritic spine density, social behavior, and special working memory were mitigated upon genetic deletion of cytoplasmic polyadenylation element-binding protein 1 (CPEB1) in *Fmr1* knockout mice. CPEB1 is an RNA-binding protein that plays a key role in post-transcriptional mRNA regulation by targeting cytoplasmic polyadenylation elements (CPEs) in the 3′ untranslated region (UTR) (Mendez and Richter, 2001; Wilczynska et al., 2005; Oe and Yoneda, 2010). The studies have concluded that excessive polypeptide elongation caused by the loss of FMRP was mitigated by the concomitant loss of CPEB1. However, due to the limitations of experimental procedures using knockout mice, the interaction between CPEB1 and *Fmr1* mRNA has not been fully analyzed.

To clarify the functional importance of CPEB1 in *Fmr1* post-transcriptional regulation, we investigated the intracellular localization and binding potential of CPEB1 and *Fmr1* mRNA in the central nervous system (CNS) neurons. In addition, we evaluated the alterations in *Fmr1* mRNA and protein levels in CPEB1 knockdown cells. We further used CPEB1 and/or FMR1 knockdown Neuro2a cells and performed microarray and gene ontology (GO) analyses to determine the cellular components and/or biological processes that might be responsible for the improvement of the *Fmr1* knockout mouse phenotypes.

## Materials and Methods

### Cell Culture and Transfection

Human cervical carcinoma HeLa cells and mouse neuroblastoma Neuro2a cells (American Type Culture Collection, Manassas, VA, United States) were cultured in Dulbecco’s modified Eagle’s medium (Thermo Fisher Scientific, Waltham, MA, United States) containing 10% fetal bovine serum (GE Healthcare, San Ramon, CA, United States). To obtain hippocampal neurons, primary cultures were performed. The animal study was reviewed and approved by the Animal Committee of Kansai Medical University. To isolate hippocampal neurons, embryonic Wistar rat brains were removed, and hippocampal regions were collected, as described previously (Banker and Cowan, 1977). Briefly, the hippocampi were collected in ice-cold Hanks’ balanced salt solution (Thermo Fisher Scientific) containing 50 μg/mL penicillin and 50 μg/mL streptomycin and then incubated in Hanks’ balanced salt solution containing 0.25% trypsin (Thermo Fisher Scientific) and 0.1% DNase I (Takara, Shiga, Japan) at 37°C for 15 min. The hippocampal tissues were gently dissociated by pipetting, centrifuged, and suspended in Neurobasal medium (Thermo Fisher Scientific) supplemented with 2% B27 (Thermo Fisher Scientific) and 0.5% GultaMAX™-I (Thermo Fisher Scientific). The cells were then plated on polyethylenimine-coated 18-mm coverslips at a concentration of 10,000 cells/mL. On the seventh day, the culture medium was replaced with fresh medium. Lipofectamine™ 2000 (Thermo Fisher Scientific) was used for transfection of plasmids into HeLa and Neuro2a cells, according to the manufacturer’s instructions. The CalPhos™ Mammalian Transfection Kit (Takara) was used for transfection of plasmids into primary hippocampal neurons, according to the manufacturer’s instructions, with previously described modifications (Oe et al., 2016). For small interfering RNA (siRNA) transfection, Stealth RNAi™ siRNAs (Thermo Fisher Scientific) targeting *Cpeb1* (accession number: NM_001252525) and *Fmr1* (accession number: NM_008031) coding regions were designed. Further, siRNA transfection was performed using Lipofectamine™ RNAiMAX (Thermo Fisher Scientific), according to the manufacturer’s instructions. Neuro2a cells were observed using a ZEISS LSM 700 confocal laser scanning microscope (Carl Zeiss, Oberkochen, Germany).

### Plasmid Construction

To construct pFmr1-3′UTR plasmid, polymerase chain reaction (PCR) was used to obtain *Fmr1* 3′UTR from C57BL/6 mouse brain cDNA (Nippon SLC, Hamamatsu, Japan) and insert it into pSL-MS2-12X. In this study, pMS2-GFP and pSL-MS2-12X (kindly provided by Dr. Robert Singer) were indicated as pGFP-MS2-NLS and pMS2rm, respectively. Expression plasmids with mutated CPEs (CPE mut 1–5) were generated using whole-vector PCR with Pfu polymerase (Promega, Madison, WI, United States). To construct mCherry-tagged CPEB1, CPEB1 cDNA coding sequence fragments were obtained from C57BL/6 mouse brain cDNA and inserted into the pmCherry-N1 vector. The oligonucleotide sequences used are listed in Supplementary Table 1.

### *In situ* Hybridization

Digoxigenin (DIG)-labeled riboprobes were designed for mouse *Fmr1* mRNA coding region (accession number: NM_008031) and synthesized using DIG RNA labeling mix (Roche Diagnostics, Basel, Switzerland), according to the manufacturer’s instructions. Frozen sections (coronal sections of hippocampus and sagittal sections of cerebellum, 20 μm thick each) of ICR mice (male, postnatal 8 weeks; Shimizu Laboratory Supplies, Kyoto, Japan) were obtained in a cryostat (CM3050S; Leica, New York, United States). *In situ* hybridization experiments using sections of the hippocampus and cerebellum were performed according to procedures described previously with slight modifications (Fujiwara et al., 2007). Briefly, sections were incubated in hybridization buffer (Roche Diagnostics) containing 250 μg/mL yeast tRNA and 100 ng/mL DIG-labeled riboprobe at 55°C for 18 h. *In situ* hybridization for cultured hippocampal neurons was performed as described previously with slight modifications (Nakagawa et al., 2014). Fourteen days *in vitro* hippocampal neurons were fixed in Ca^2+^- and Mg^2+^-free saline buffered with 4-(2-hydroxyethyl)-1-piperazineethanesulfonic acid (10 mM, pH 7.4) containing 4% paraformaldehyde at 37°C for 15 min and permeabilized with phosphate-buffered saline (PBS) containing 0.1% Triton™ X-100 at 25°C for 5 min. Neurons were then prehybridized in hybridization buffer (Roche Diagnostics) containing 250 μg/mL yeast tRNA at 55°C for 2 h. Hybridization was performed for 18 h at 55°C in hybridization buffer containing 100 ng/mL DIG-labeled riboprobe. Excessive riboprobes were removed by serial washing steps; 2 × SSC buffer containing 50% formamide at 55°C, 2 × 15 min each time, 2 × SSC buffer at 55°C for 15 min, 0.2 × SSC buffer at 55°C, 2 × 15 min each time. Subsequent immunostaining was performed using an anti-DIG mouse monoclonal antibody (used at a dilution of 1:500, AB_2339005; Jackson ImmunoResearch, West Grove, PA, United States), anti-CPEB1 rabbit polyclonal antibody (1:500 dilution, ab73287, Abcam, Cambridge, United Kingdom), and anti-microtubule-associated protein 2 (MAP2) chicken polyclonal antibody (1:2,000 dilution, ab5392, Abcam) at 25°C for 1 h. The coverslips were then incubated with Alexa Fluor 594-labeled donkey anti-mouse immunoglobulin (Ig) G (1:500 dilution, 715-585-151, Jackson ImmunoResearch), Alexa Fluor 488-labeled donkey anti-rabbit IgG (1:1,000 dilution; A21206; Molecular Probes, Eugene, OR, United States), and Alexa Fluor 647-labeled donkey anti-chicken IgY (1:2,000 dilution, 703-605-155, Jackson ImmunoResearch) at 4°C for 1 h. Fluorescence images were obtained using a ZEISS LSM 700 confocal laser scanning microscope (Carl Zeiss). Although experiments were adequately performed, slight non-specific signals caused by secondary antibodies were observed in sense probe images.

### Immunofluorescence Microscopy

Cells were fixed in PBS containing 4% paraformaldehyde at 37°C for 15 min and permeabilized with PBS containing 0.1% Triton™ X-100 at 25°C for 5 min. After blocking with PBS containing 3% bovine serum albumin at 25°C for 1 h, the cells were incubated with an anti-heat shock protein family A member 9 (HSPA9) rabbit polyclonal antibody (1:500 dilution, RB-10470-P0, Thermo Fisher Scientific), anti-FMRP rabbit polyclonal antibody (1:500 dilution, ab17722, Abcam), and anti-MAP2 chicken polyclonal antibody (1:2,000 dilution, ab5392, Abcam) for 24 h at 4°C. The cells were then incubated with Alexa Fluor 488-labeled donkey anti-rabbit IgG (1:1,000 dilution, A21206, Molecular Probes) and Alexa Fluor 647-labeled donkey anti-chicken IgY (1:2,000 dilution, 703-605-155, Jackson ImmunoResearch) at 25°C for 1 h. After mounting with a solution containing Hoechst 33258, the cells were observed using a ZEISS LSM 700 confocal laser scanning microscope (Carl Zeiss).

### Quantitative PCR

For Quantitative PCR (qPCR), total RNA was collected from Neuro2a cells using TRIzol™ reagent (Thermo Fisher Scientific). Further, cDNA synthesis was performed using the PrimeScript™ RT Reagent Kit with gDNA Eraser (Takara) according to the manufacturer’s instructions. Moreover, qPCR was performed using THUNDERBIRD™ SYBR^®^ qPCR Mix (TOYOBO, Osaka, Japan) and the Rotor-Gene^®^ Q real-time PCR system (Qiagen, Hilden, Germany). Hypoxanthine guanine phosphoribosyl transferase was used as an internal control. The oligonucleotide sequences used are listed in Supplementary Table 1.

### Sodium Dodecyl Sulfate-Polyacrylamide Gel Electrophoresis and Western Blot Analysis

For Sodium Dodecyl Sulfate-Polyacrylamide Gel Electrophoresis (SDS-PAGE) sample preparation, Neuro2a cells were lysed using a buffer containing 50 mM Tris HCl (pH 7.5), 0.1% SDS, and 1 mM dithiothreitol. Twenty-microgram samples were electrophoresed on 10% polyacrylamide gels and transferred to polyvinylidene fluoride membranes. The primary antibodies used in this study were rabbit polyclonal antibodies against CPEB1 (ab73287, Abcam), FMRP (ab617722, Abcam), and glyceraldehyde-3-phosphate dehydrogenase (GAPDH; sc-25778, Santa Cruz Biotechnology, Santa Cruz, CA, United States). The secondary antibody was horseradish peroxidase-conjugated donkey anti-rabbit IgG (H + L) antibody (Jackson ImmunoResearch). Primary antibodies and horseradish peroxidase-conjugated secondary antibodies were used at dilutions of 1:500 and 1:1,000, respectively, in 3% bovine serum albumin in Tris-buffered saline containing 0.1% Tween^®^ 20. The proteins were visualized using Luminata Classico Western Horseradish Peroxidase Substrate (Millipore, Burlington, MA, United States) and detected using ImageQuant™ LAS 4000 mini (GE Healthcare).

### RNA Immunoprecipitation Assay

RNA Immunoprecipitation (RNA-IP) assays were performed as previously described (Alspach and Stewart, 2015). RNA was immunoprecipitated using CPEB1 (ab73287, Abcam), FMRP (ab617722, Abcam), GAPDH (sc-25778, Santa Cruz Biotechnology), and normal rabbit IgG (#2729, Cell Signaling Technology, Danvers, MA, United States) antibodies. Further, cDNA was synthesized from the precipitated RNA using the SuperScript™ III First-Strand Synthesis System (Thermo Fisher Scientific). The *Fmr1* mRNA cDNA in each immunoprecipitation product was detected using PCR with KOD plus (TOYOBO), and the product was separated using 1% agarose gel electrophoresis. For qPCR, cDNA was synthesized from the immunoprecipitated RNAs using the PrimeScript RT Reagent Kit with gDNA Eraser (TaKaRa). Subsequent qPCR was performed using THUNDERBIRD SYBR qPCR Mix (TOYOBO) and Rotor-Gene Q Real-time PCR System (Qiagen).

### Microarray and Gene Ontology Analyses

Total RNA was extracted from Neuro2a cells transfected with each siRNA. The RNAs were sent to Riken Genesis (Tokyo, Japan) for microarray and GO analyses. Briefly, RNA quality was examined using an Agilent 2100 Bioanalyzer (Agilent Technologies, Santa Clara, CA, United States). Microarray experiments were performed using the Affymetrix GeneChip^§^ Mouse Genome 430 2.0 Array (Affymetrix, Santa Clara, CA, United States). Microarray data were submitted to the Gene Expression Omnibus (GEO) and assigned GEO accession number as GSE147604.

### Statistical Analysis

Data are presented as the mean ± standard deviation of at least three independent experiments. Statistical analysis was performed using one-way analysis of variance followed by the Bonferroni–Dunn test. Significant values (*P*) are indicated in figure legends.

## Results

### Cytoplasmic Polyadenylation Element-Binding Protein 1 Co-localized With Fragile X Mental Retardation 1 mRNA in Neurons

**FIGURE 1:**
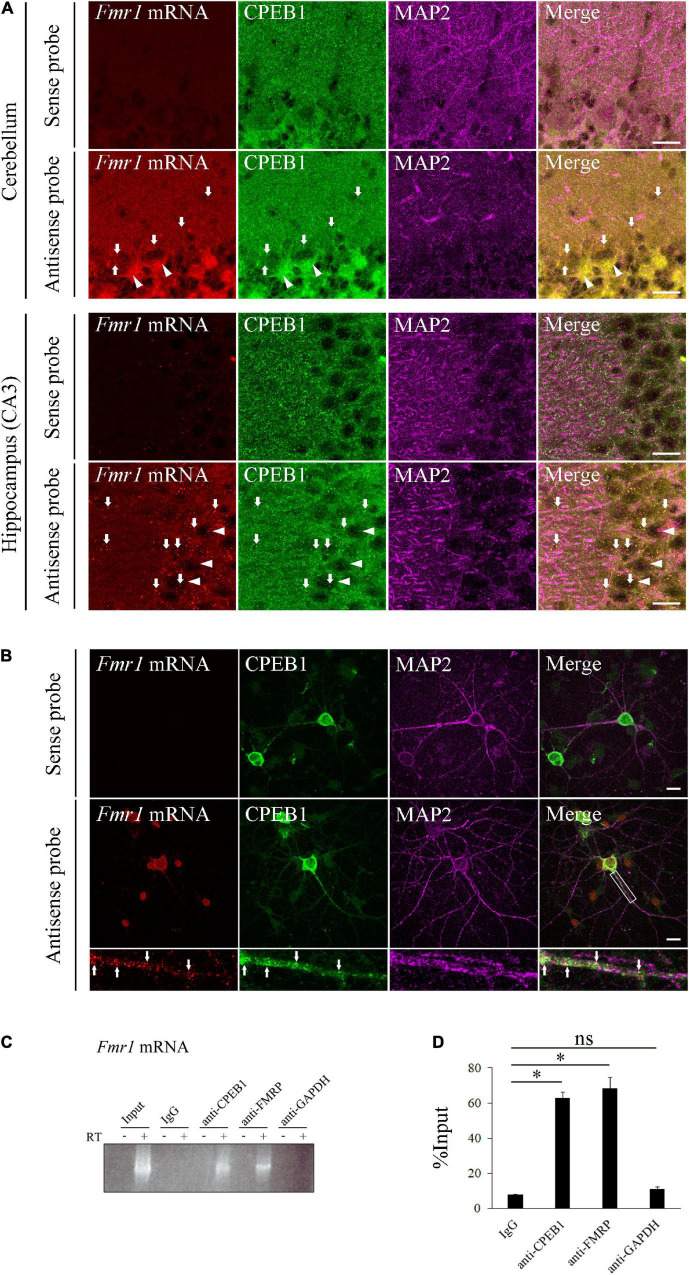
Cytoplasmic polyadenylation element-binding protein 1 (CPEB1) co-localizes and interacts with fragile X mental retardation 1 (*Fmr1*) mRNA in neurons. **(A)** CPEB1 and *Fmr1* mRNA localization in the cerebellum and hippocampus was observed using *in situ* hybridization and simultaneous immunostaining. Arrows show examples of CPEB1-*Fmr1* mRNA co-localization. Arrowheads show representative Purkinje cells in the cerebellum and pyramidal cells in the hippocampus. **(B)** CPEB1 and *Fmr1* mRNA intracellular localization in hippocampal neurons was observed using *in situ* hybridization and simultaneous immunostaining. Fluorescence signals in white boxes are shown in enlarged images. Arrows in enlarged images show examples of CPEB1-*Fmr1* mRNA co-localization. **(C)** Endogenous *Fmr1* mRNA detection using reverse transcriptase polymerase chain reaction (RT-PCR) following immunoprecipitation. *Fmr1* mRNA was analyzed performing RT-PCR on mouse brain lysates (Input) and the immunoprecipitation products obtained from the mouse brain lysates using CPEB1, fragile mental retardation protein (FMRP), and glyceraldehyde-3-phosphate dehydrogenase (GAPDH) antibodies. **(D)** *Fmr1* mRNA levels in the immunoprecipitation products were analyzed using RNA immunoprecipitation assay with indicated antibodies and subsequent quantitative polymerase chain reaction (qPCR) (*n* = 4). Statistical analysis was performed using one-way analysis of variance followed by the Bonferroni–Dunn test. ns indicates not significant, *indicates *P* < 0.05. Scale bar; 20 μm.

The molecular interaction between CPEB1 and *Fmr1* mRNA has not been investigated; however, a previous study has shown that CPEB1 and *Fmr1* mRNA individually exist in cerebellar and hippocampal neurons (Ferrari et al., 2007; McEvoy et al., 2007). To determine whether CPEB1 regulates *Fmr1* mRNA dynamics, we first used *in situ* hybridization to demonstrate CPEB1 and endogenous *Fmr1* mRNA localizations in the hippocampal and cerebellum, and primary hippocampal neurons (Figures 1A,B). In the cerebellum, *Fmr1* mRNA was predominantly observed in the cell body of Purkinje cells and co-localized with CPEB1 (Figure 1A, arrows). In the hippocampus, *Fmr1* mRNA was observed in the cell body and MAP2-positive dendrites of CA3 neurons and co-localized with CPEB1 (Figure 1A, arrows). In cultured hippocampal neurons, *Fmr1* mRNA was observed in the cell body and MAP2-positive dendrites in a granular manner, and often co-localized with CPEB1 (Figure 1B, arrows). Following RNA-IP on mouse brain lysates using antibodies against CPEB1 and FMRP, reverse transcriptase PCR (RT-PCR) was performed to confirm the binding of CPEB1 and *Fmr1* mRNA. CPEB1 bound to *Fmr1* mRNA, whereas IgG and GAPDH antibodies did not (Figure 1C). Consistent with a previous study (Ferrari et al., 2007), FMRP also bound to *Fmr1* mRNA (Figure 1C). Furthermore, we performed qPCR following an RNA-IP assay to confirm the interaction between CPEB1 and *Fmr1* mRNA in mouse brain lysates (Figure 1D). We revealed that endogenous *Fmr1* mRNA pulled down by CPEB1 or FMRP antibody was significantly enriched compared with that in IgG and GAPDH antibody immunoprecipitation products. These results suggested that CPEB1 interacted with endogenous *Fmr1* mRNA in central nervous system (CNS) neurons.

### Cytoplasmic Polyadenylation Element-Binding Protein 1 Co-localized With Fragile X Mental Retardation 1 3′Untranslated Region in HeLa Cells

**FIGURE 2:**
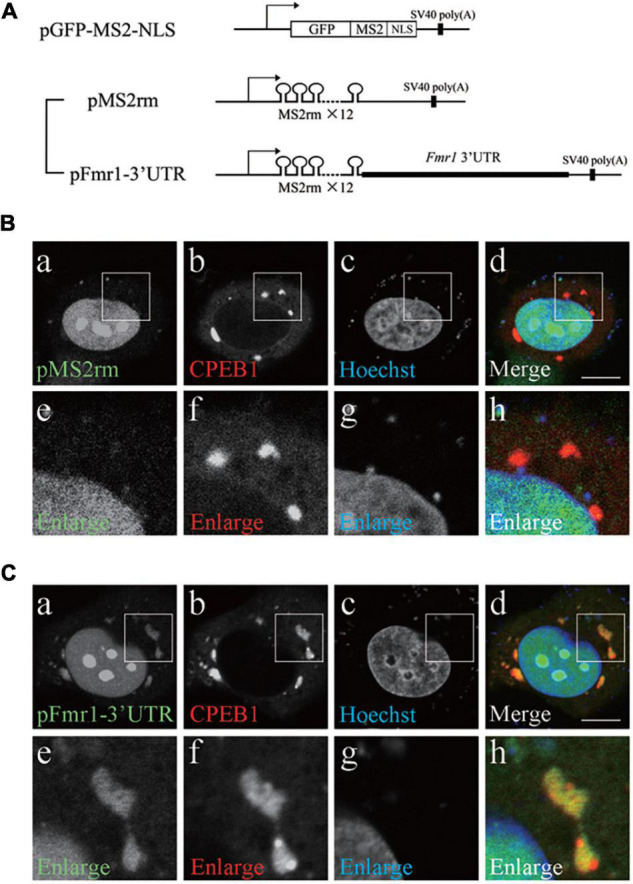
Cytoplasmic polyadenylation element-binding protein 1 (CPEB1) and fragile X mental retardation 1 (*Fmr1)* 3′ untranslated region (UTR) co-localization in HeLa cells. **(A)** Schematic representation of the MS2-based mRNA visualization procedure. Plasmid pGFP-MS2-NLS expressed a bacteriophage MS2 coat protein fused with green fluorescent protein (GFP) and nuclear localization signal (NLS). Plasmid pMS2rm expressed mRNA containing twelve tandem repeats of MS2 recognition motif (MS2rm). Plasmid pFmr1-3′UTR expressed mRNA containing *Fmr1* 3′UTR downstream of MS2rm. **(B)** HeLa cells were transfected with pGFP-MS2-NLS, pmCherry-CPEB1, and pMS2rm **(a–d)**. Boxed insets are enlarged **(e–h)**. **(C)** HeLa cells were transfected with pGFP-MS2-NLS, pmCherry-CPEB1, and pFmr1-3′UTR **(a–d)**. Boxed insets are enlarged **(e–h)**. Scale bar; 20 μm.

Next, we examined the significance of *Fmr1* 3′UTR in the interaction between CPEB1 and *Fmr1* mRNA. We constructed a pFmr1-3′UTR expression plasmid using an MS2-based mRNA visualization system (Figure 2A). The pGFP-MS2-NLS plasmid expresses a bacteriophage MS2 coat protein, which is fused with green fluorescent protein (GFP) and nuclear localization signal (NLS), recognizing tandem repeats of MS2 recognition motif (MS2rm). GFP-MS2-NLS localization in the cytoplasm represented the site of MS2rm-containing mRNA expressed by pMS2rm and pFmr1-3′UTR plasmids, whereas the remaining GFP-MS2-NLS that did not interact with MS2rm-containing mRNA was localized to the nucleus. We transfected the pMS2rm or pFmr1-3′UTR plasmid, in addition to the pGFP-MS2-NLS and pmCherry-CPEB1 plasmids, into HeLa cells, a commonly used CPEB1-expressing cell line (Serman et al., 2007). In pMS2rm-transfected cells, GFP was observed in the nucleus but not in the cytoplasm, whereas mCherry-tagged CPEB1 formed small foci and large granules in the cytoplasm (Figures 2Ba–h). In contrast, in pFmr1-3′UTR-transfected cells, GFP-positive granules were observed in the cytoplasm and co-localized with CPEB1-positive granules (Figures 2Ca–h). Previous studies have demonstrated that CPEB1 acts as a major regulator of stress granule (mRNA-ribonucleoprotein complex) formation, and modulates translational efficiency under stress conditions, including heat shock (Namkoong et al., 2018), hypoxia (Arimoto et al., 2008), and viral infection (McCormick and Khaperskyy, 2017). These results suggested that CPEB1 interacted with *Fmr1* mRNA through the 3′UTR and incorporated *Fmr1* mRNA into the mRNA-ribonucleoprotein complex in HeLa cells.

### Cytoplasmic Polyadenylation Element Mutation in Fragile X Mental Retardation 1 3′Untranslated Region Reduced the Efficiency of Localization to Cytoplasmic Polyadenylation Element-Binding Protein 1-Positive Granules

**FIGURE 3:**
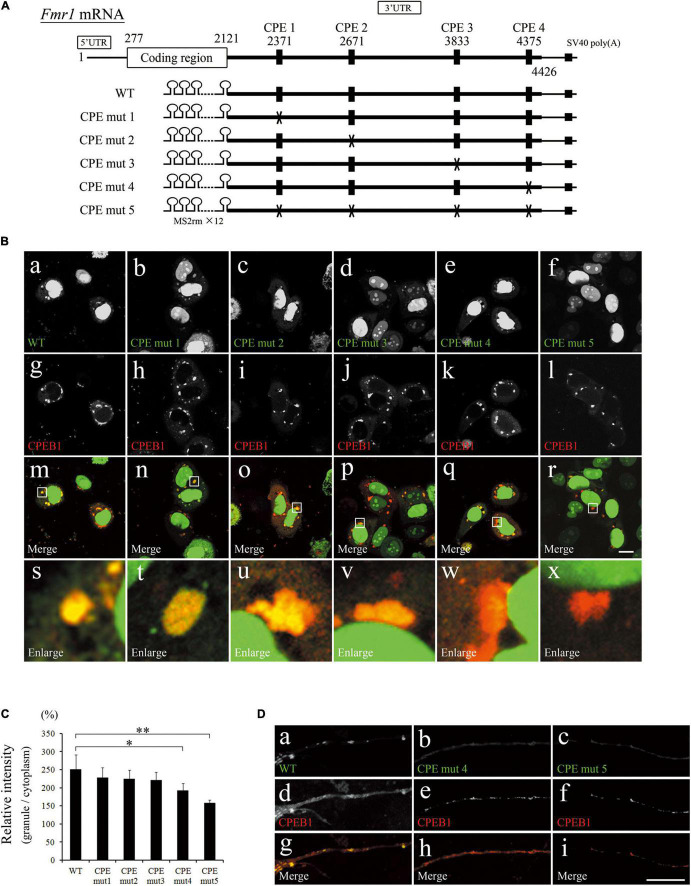
Cytoplasmic polyadenylation element (CPE) mutations repressed localization of fragile X mental retardation 1 (*Fmr1*) 3′ untranslated region (UTR) to cytoplasmic polyadenylation element-binding protein 1 (CPEB1)-positive granules. **(A)** Schematic representation of *Fmr1* mRNA and *Fmr1* 3′UTR-expressing plasmids. **(B)** *Fmr1* 3′UTR **(a–f)** and mCherry-tagged CPEB1 **(g–l)** subcellular localizations in HeLa cells transfected with the indicated plasmids. Enlarged versions **(s–x)** of the white boxes are shown in the merged images **(m–r)**. **(C)** Quantification of localization ratio of *Fmr1* 3′UTR to CPEB1-positive granules. Green fluorescent protein (GFP) fluorescence intensity in CPEB1-positive granules was measured and compared with that of cytoplasmic GFP. At least 96 CPEB1-positive granules were measured for each expression plasmid. **(D)** *Fmr1* 3′UTR **(a–c)** and mCherry-tagged CPEB1 **(d–f)** intracellular localization in hippocampal neurons transfected with the indicated plasmids (**g–i**, merged images). Scale bar: 15 μm. Statistical analysis was performed using one-way ANOVA followed by the Bonferroni–Dunn test. *indicates *P* < 0.05, ^**^indicates *P* < 0.01.

Nucleotide sequence search revealed that *Fmr1* 3′UTR contained four CPEs that could be recognized by CPEB1 (Figure 3A). To clarify whether CPEB1 interacted with *Fmr1* 3′UTR in a CPE-dependent manner, we constructed expression plasmids with mutated CPEs (Figure 3A). GFP and mCherry-tagged CPEB1 intracellular localizations were observed in HeLa cells transfected with the indicated plasmids (Figures 3Ba–x). Intense GFP signals on CPEB1-positive granules were observed in pFmr1-3′UTR-transfected cells (indicated as WT in Figure 3A), whereas weak GFP signals were observed in CPE-mut5-transfected cells. To quantify the efficiency of GFP localization in CPEB1-positive granules, relative intensity was calculated by comparing the GFP fluorescence intensity of CPEB1-positive granules with diffuse GFP intensity in the cytoplasm (Figure 3C). In CPE- mut1-, CPE- mut2-, and CPE-mut3-transfected cells, the localization efficiency was approximately equal to that in pFmr1-3′UTR-transfected cells (indicated as WT in Figure 3A); however, a significant reduction in the efficiency of localization was observed in CPE-mut4- and CPE-mut5-transfected cells (Figure 3C). Next, we attempted to confirm the effects of CPE mutations in primary hippocampal neurons. GFP co-localized with CPEB1-positive granules in pFMR1-3′UTR-transfected neuronal dendrites, whereas GFP was diffused and did not co-localize with CPEB1-positive granules in CPE-mut4- and CPE-mut5-transfected neuronal dendrites (Figure 3D). These findings suggest that CPEB1 interacts with *Fmr1* mRNA in a CPE-dependent manner for its localization to the mRNA-ribonucleoprotein complex.

### Cytoplasmic Polyadenylation Element-Binding Protein 1 Knockdown Upregulated Fragile X Mental Retardation 1 mRNA and Protein Levels and Altered Fragile Mental Retardation Protein Intracellular Localization

**FIGURE 4:**
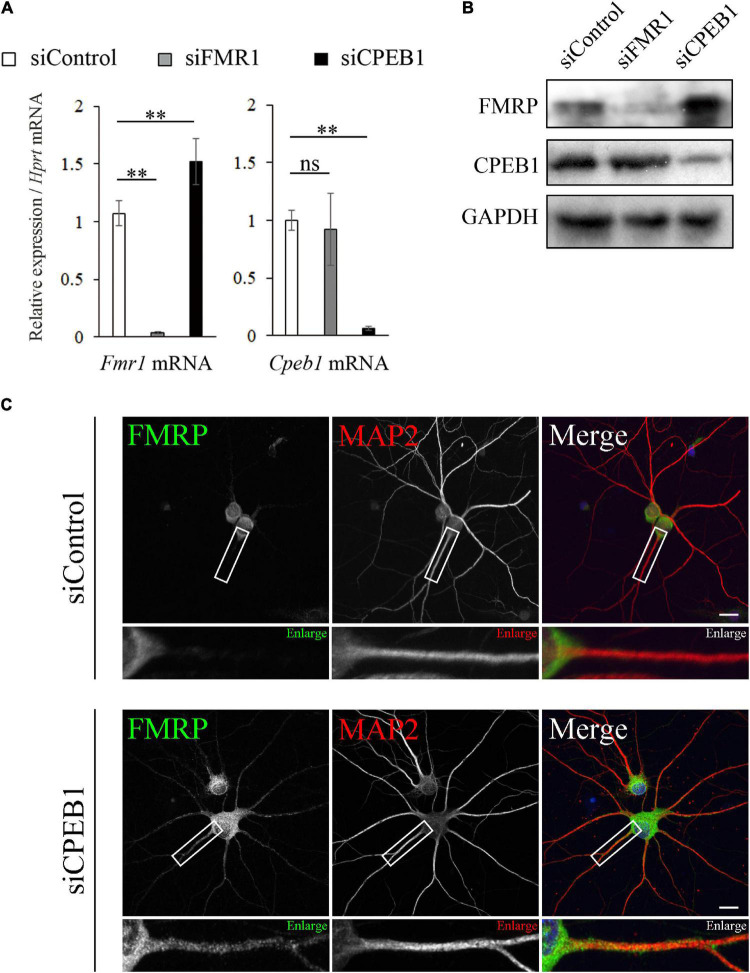
Cytoplasmic polyadenylation element-binding protein 1 (CPEB1) knockdown upregulates fragile X mental retardation 1 (*Fmr1*) mRNA and protein levels and alters fragile mental retardation protein (FMRP) intracellular localization in hippocampal neurons. **(A)** Relative *Cpeb1* and *Fmr1* mRNA levels in siControl-, siFMR1-, and siCPEB1-treated Neuro2a cells were analyzed using qPCR (*n* = 4). **(B)** FMRP, CPEB1, and glyceraldehyde-3-phosphate dehydrogenase (GAPDH) protein levels in siControl-, siFMR1-, and siCPEB1-treated Neuro2a cells were detected using western blot. **(C)** FMRP intracellular localization in siControl- and siFMR1-treated hippocampal neurons were observed using immunostaining. Boxed insets are enlarged. Statistical analysis was performed using one-way analysis of variance, followed by the Bonferroni–Dunn test. ns indicates not significant, ^**^indicates *P* < 0.01. Scale bar; 20 μm.

Considering the CPE-dependent interaction between CPEB1 and *Fmr1* mRNA, we hypothesized that CPEB1 post-transcriptionally regulates *Fmr1* mRNA translation. Therefore, we performed siRNA-mediated knockdown experiments using Neuro2a cells, a commonly used FMRP and CPEB1-expressing neuronal cell line (Zhou et al., 2017; Oe et al., 2020). Transfection of specific siRNAs targeting *Fmr1* mRNA (siFMR1) or *Cpeb1* mRNA (siCPEB1) robustly and specifically reduced target gene mRNA and protein levels in Neuro2a cells compared with those in control siRNA (siControl)-transfected cells (Figures 4A,B). Notably, *Fmr1* mRNA and protein expression levels were obviously upregulated in siCPEB1-transfected cells compared with those in siControl-treated cells. In contrast, *Cpeb1* mRNA and protein levels were not affected in siFMR1-transfected cells. We next examined whether CPEB1 knockdown affected FMRP dynamics in primary hippocampal neurons (Figure 4C). In siControl-treated neurons, FMRP was observed in the cell body and proximal region of MAP2-positive dendrites. In contrast, FMRP was found in the cell body and further expanded to the distal region of siCPEB1-treated neuronal dendrites (Figure 4C). These findings suggested that CPEB1 post-transcriptionally repressed *Fmr1* mRNA and protein levels in Neuro2a cells. Furthermore, CPEB1-dependent FMRP expression regulation might be required for the accurate FMRP localization in neurons.

### Cytoplasmic Polyadenylation Element-Binding Protein 1 Knockdown Altered Expression Levels of Numerous Genes in Fragile X Mental Retardation 1 Knockdown Neuro2a Cells

A previous study has demonstrated that genetic depletion of CPEB1 ameliorates behavioral abnormalities in *Fmr1* knockout mice, an experimental FXS model (Udagawa et al., 2013). However, it is impossible to investigate the interaction between CPEB1 and *Fmr1* mRNA in *Cpeb1*/*Fmr1* double-knockout mice. We therefore performed microarray analysis using CPEB1 and/or FMR1 knockdown cells to provide a comprehensive evaluation of gene expression alteration in FXS cell model (Supplementary Tables 2–7). Compared to siControl-transfected cells, siCPEB1-transfected cells expressed 650 genes that showed more than twofold upregulation and 808 genes that were downregulated to less than half. Furthermore, compared to siControl-transfected cells, there were 250 upregulated genes and 158 downregulated genes in siFMR1-transfected cells, a widely used FXS cell model (Korb et al., 2017). Moreover, compared to siFMR1-transfected cells, there were 464 genes that showed more than twofold upregulation and 612 genes that were downregulated to less than half in siFMR1/siCPEB1-transfected cells. Notably, our data showed overlapping genes between siCPEB1/siControl- and siFMR1/siControl-transfected cells: 87 upregulated and 38 downregulated genes. Accordingly, 30.6% (125/408) of genes, expression levels of which were affected by FMR1 knockdown, also showed altered expression due to CPEB1 knockdown. These results are supported by bioinformatic data showing that approximately one-third of FMRP-targeted mRNAs are potential targets for CPEB1 (Udagawa et al., 2013). Furthermore, we performed GO analysis using microarray results (Supplementary Table 8). We found that a subset of genes annotated with mitochondria-related GO terms was upregulated in siFMR1/siCPEB1-transfected cells compared with that in siFMR1-transfected cells. These findings suggested that CPEB1 knockdown altered the expression levels of numerous genes, including mitochondria-related genes, in FMR1 knockdown cells.

### Cytoplasmic Polyadenylation Element-Binding Protein 1 Knockdown Upregulated mRNA Levels of Mitochondria-Related Genes and Restored Intracellular HSPA9 Localization

**FIGURE 5:**
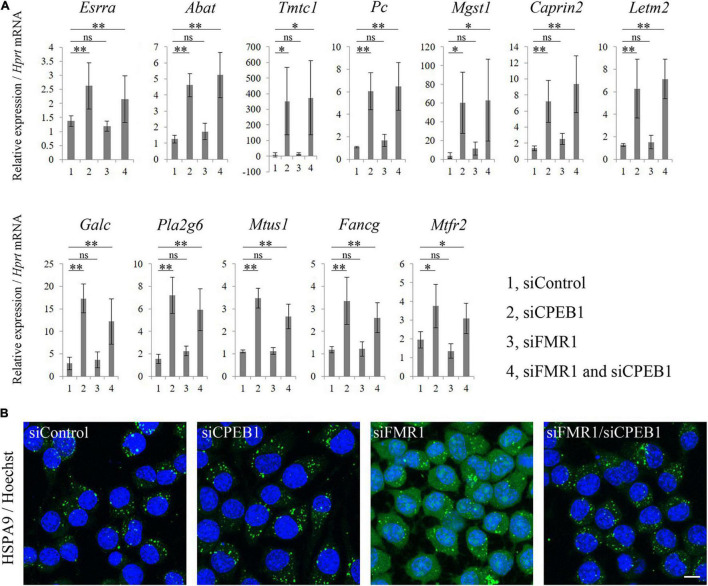
Cytoplasmic polyadenylation element-binding protein 1 (CPEB1) knockdown upregulates mitochondria-related mRNA levels and rescues aberrant heat shock protein family A member 9 (HSPA9) localization in fragile X mental retardation 1 (FMR1) knockdown cells. **(A)** Quantitative polymerase chain reaction (qPCR) analysis of mitochondria-related mRNAs identified using microarray analysis (*n* = 4). **(B)** Immunofluorescence images of HSPA9 in Neuro2a cells treated with the indicated siRNAs. Anti-HSPA9 antibody showed aberrant localization, that is a more diffused distribution, in FMR1 knockdown cells compared with that in control cells, and CPEB1 knockdown ameliorated HSPA9 localization in FMR1/CPEB1 knockdown cells. Scale bar: 15 μm. Statistical analysis was performed using one-way analysis of variance, followed by Bonferroni–Dunn test. ns indicates not significant, *indicates *P* < 0.05, ^**^indicates *P* < 0.01.

Based on the GO analysis results, we performed qPCR analysis using total RNA from siControl-, siCPEB1-, siFMR1-, and siFMR1/siCPEB1-transfected Neuro2a cells. We examined the substantial mRNA levels of genes that were annotated with mitochondria-related terms in siFMR1/siCPEB1-transfected cells, compared with those in siFMR1-transfected cells. These genes included estrogen-related receptor alpha (*Esrra*), 4-aminobutyrate aminotransferase (*Abat*), transmembrane O-mannosyltransferase-targeting cadherin 1 (*Tmtc1*), pyruvate carboxylase (*Pc*), microsomal glutathione S-transferase 1 (*Mgst1*), caprin family member 2 (*Caprin2*), leucine zipper and EF-hand-containing transmembrane protein 2 (*Letm2*), galactosylceramidase (*Galc*), phospholipase A2 group VI (*Pla2g6*), microtubule associated scaffold protein 1 (*Mtus1*), FA complementation group G (*Fancg*), and mitochondrial fission regulator 2 (*Mtfr2*) (Figure 5A). The mRNA expression levels of these genes were not altered in siFMR1-transfected cells compared with those in siControl-transfected cells. However, the mRNA levels were significantly upregulated in siCPEB1- and siFMR1/siCPEB1-transfected cells compared with those in siControl-transfected cells (Figure 5A). Moreover, we performed immunofluorescence microscopy to identify the intracellular localization of HSPA9, a mitochondrial chaperone protein that plays important roles in mitochondrial protein quality control, mitochondrial morphology, and apoptosis inhibition (Geissler et al., 2001; Yang et al., 2008; Burbulla et al., 2010). We observed that HSPA9 formed numerous dot-like structures in siControl-transfected cells (Figure 5B). HSPA9 intracellular localization was not altered in siCPEB1-transfected cells compared to that in siControl-transfected cells. Interestingly, in addition to dot-like structures, siFMR1-transfected cells showed a diffused HSPA9 distribution compared with that siControl-transfected cells. Notably, the aberrant HSPA9 localization in siFMR1-transfected cells was restored in siFMR1/siCPEB1-treated cells (Figure 5B). These results suggested that CPEB1 was involved in mitochondrial functions through modulation of the mRNA expression levels of mitochondria-related genes and HSPA9 localization in FMR1 knockdown cells.

## Discussion

In this study, we reported two major findings regarding the roles of CPEB1 under physiological conditions and in the FXS cell model: (i) CPEB1 controlled *Fmr1* mRNA intracellular localization and post-transcriptionally regulated *Fmr1* expression, and (ii) CPEB1 was involved in mitochondrial functions through modulation of the mRNA expression levels of mitochondria-related genes and HSPA9 localization in FMR1 knockdown Neuro2a cells.

First, we demonstrated that CPEB1 controlled *Fmr1* mRNA intracellular localization and post-transcriptionally regulated *Fmr1* expression. CPEB1 co-localized and interacted with *Fmr1* 3′UTR in the cytoplasmic granular compartment (Figures 1–3). In addition, CPEB1 was considered to repress *Fmr1* mRNA and protein levels (Figures 4A,B). Following immunoprecipitation, we used an MS2-based RNA visualization system and RT-PCR and revealed that CPEB1 interacted with *Fmr1* mRNA by recognizing CPEs in the 3′UTR and recruited *Fmr1* mRNA into mRNA-ribonucleoprotein complexes. In general, CPEB1 recognizes CPEs in the 3′UTR and represses polyadenylation and CPE-containing mRNA translation. CPEB1 phosphorylation inhibits repression of polyadenylation and subsequent translation (Mendez and Richter, 2001). CPEB1-dependent translational regulation occurs in granular mRNA-ribonucleoprotein complexes, known as stress or RNA granules. Under various environmental stresses, including heat shock, ultraviolet radiation, hypoxia, and virus infection, stress granules emerge, repress overall translational activity, reduce cellular energy consumption, and contribute to increased cell survival (Panas et al., 2016). In neuronal dendrites, RNA granules act as transport machineries that bi-directionally move along cytoskeletal structures such as microtubules in a translational-dormant state and resume translation in response to synaptic stimulation in the post-synaptic region. Indeed, previous studies have shown that *Fmr1* mRNA and CPEB1 each localize to RNA granules and are transported to the postsynaptic region in dendrites (Ferrari et al., 2007; Ohashi and Shiina, 2020). Considering that synaptic activity-induced CPEB1 phosphorylation de-represses and resumes CPE-containing mRNA translation in RNA granules (Baj et al., 2016), our data suggested that CPEB1 interacted with *Fmr1* mRNA through the 3′UTR of the granular mRNA-ribonucleoprotein complex, such as stress and RNA granules, and regulated *Fmr1* mRNA transport and translation in neuronal dendrites.

Second, we demonstrated that CPEB1 was involved in mitochondrial functions through modulation of the mRNA expression levels of mitochondria-related genes and HSPA9 localization. Using microarray and GO analyses, we revealed that CPEB1 knockdown alters the mRNA expression levels of numerous genes in the control and FMR1 knockdown cells widely used as an FXS cell model (Korb et al., 2017). In particular, CPEB1 knockdown increased the mRNA levels of genes annotated with mitochondria-related GO terms (Figure 5A). Some of these mitochondria-related genes play important roles in mitochondrial functions: (i) mitochondrial metabolic functions, including nucleoside metabolism, calcium homeostasis, and antioxidant activity, are regulated by *Abat* (Besse et al., 2015), *Tmtc1* (Sunryd et al., 2014), *Pc* (Cappel et al., 2019), *Mgst1* (Johansson et al., 2010), and *Fancg* (Mukhopadhyay et al., 2006) protein products; and (ii) mitochondrial morphology is controlled by *Esrra* (Singh et al., 2018), *Letm2* (Tamai et al., 2008), *Mtus1* (Wang et al., 2018), and *Mtfr2* (Lu et al., 2019) protein products. Interestingly, our data showed that CPEB1 knockdown significantly increased the mRNA levels of *Pla2g6*, which has been identified as the causative gene for an autosomal recessive form of Parkinson’s disease (Gregory et al., 2008). Considering that *Pla2g6* protein product acts as an A2 phospholipase and plays key roles in membrane remodeling through phospholipid deacylation and reacylation cycle (Mori et al., 2019), our results implied that CPEB1 was involved in multiple mitochondrial functions, including metabolism, morphology, and membrane remodeling.

Several lines of evidence have indicated mitochondrial dysfunction, such as abnormal morphology, respiration rate, and ATP production, in FXS mouse models (D’Antoni et al., 2020; Kuzniewska et al., 2020). Our data, which showed an aberrant HSPA9 distribution in FMR1 knockdown cells, might reflect mitochondrial dysfunction consistent with that in the FXS models. A previous study has shown that HSPA9 overexpression exerts a protective effect against complement-dependent cytotoxicity in erythroleukemia cells; however, HSPA9 truncated mutant, which lacks a mitochondrial-targeting signal peptide, when overexpressed, is unable to exert the same effect (Saar Ray et al., 2014). Thus, we consider that the improved HSPA9 distribution observed in siFMR1/siCPEB1-treated cells represents amelioration of mitochondrial function.

In FXS pathology, structural and functional deficits have been observed in the cerebral cortex, hippocampus, and cerebellum (Huber, 2006; Mercaldo et al., 2009; Guo et al., 2012). Previous studies demonstrated that loss of FMRP in the cerebellum exhibits aberrant morphology of synaptic mitochondria, abnormally elongated spines in Purkinje cells, enhanced long-term depression (LTD) at the parallel fiber synapses that innervate these spines, and deficits in motor learning (Koekkoek et al., 2005). Indeed, FXS patients displays abnormal performance in the acquisition of conditioned eyelid responses compared with normal individuals (Smit et al., 2008). These studies suggest that FMRP plays pivotal roles in physiological structure and synaptic plasticity in the cerebellum. Additionally, several lines of evidence suggest that CPEB1 is also important for cerebellar function; abnormally increased spine density, longer spines, abolished protein synthesis-dependent LTD, and ataxia were observed in the cerebellum in mice expressing CPEB1-AA that is a phosphorylation mutant and defective in exerting polyadenylation mediated-translation (McEvoy et al., 2007). Considering that reduction of mitochondrial ATP production was observed in hippocampal neurons in CPEB1 KO mice (Oruganty-Das et al., 2012), CPEB1 might be involved in mitochondrial function in the cerebellum. Together, it is plausible that mitochondrial function, spine morphology, synaptic plasticity, motor learning in the cerebellum could be regulated by FMRP and/or CPEB1, both of which regulate synaptic local translation and contribute to translational homeostasis in neurons.

Here, we have shown that CPEB1 is co-localized with *Fmr1* mRNA in hippocampal neurons and cerebellar Purkinje cells and post-transcriptionally regulates FMRP expression. Moreover, loss of CPEB1 might ameliorate mitochondrial functions through regulating mitochondrial gene expression and modulating HSP9 localization in FMR1 knockdown cells. It is thus possible that physiological neuronal functions are maintained by mitochondrial activity which is regulated through CPEB1/FMRP cascade not only in the hippocampus but also in the cerebellum.

Finally, our data suggested that CPEB1 post-transcriptionally repressed *Fmr1* expression through the 3′UTR, and that CPEB1 knockdown might affect mitochondrial function. Further studies are required to elucidate the detailed mitochondrial functions of CPEB1 using CPEB1 knockdown FXS model.

## Data Availability Statement

The datasets presented in this study can be found in online repositories. The names of the repository/repositories and accession number(s) can be found below: https://www.ncbi.nlm.nih.gov/geo/query/acc.cgi?acc=GSE147604.

## Ethics Statement

The animal study was reviewed and approved by the Animal Committee of Kansai Medical University.

## Author Contributions

MK, HY, and YAN conceived and supervised the study. SO, RS-O, and TK designed the experiments. SO, RK, and SS performed the experiments. SO, YON, and YH analyzed the data. SO, SH, and ST wrote the manuscript. All authors read and approved the final manuscript.

## Conflict of Interest

The authors declare that the research was conducted in the absence of any commercial or financial relationships that could be construed as a potential conflict of interest.

## Publisher’s Note

All claims expressed in this article are solely those of the authors and do not necessarily represent those of their affiliated organizations, or those of the publisher, the editors and the reviewers. Any product that may be evaluated in this article, or claim that may be made by its manufacturer, is not guaranteed or endorsed by the publisher.

## Abbreviations

CNS: central nervous system
CPEB1: cytoplasmic polyadenylation element-binding protein 1
Fmr1: fragile X mental retardation 1
FMRP: fragile mental retardation protein
UTR: untranslated region
FXS: fragile X syndrome
CPE: cytoplasmic polyadenylation element
GO: gene ontology
DIG: digoxigenin
Ig: immunoglobulin
PBS: phosphate-buffered saline
HSPA9: heat shock protein family A member 9
RT-PCR: reverse-transcriptase polymerase chain reaction
MAP2: microtubule-associated protein 2
SDS-PAGE: sodium dodecyl sulfate-polyacrylamide gel electrophoresis
siRNA: small interfering RNA
GAPDH: glyceraldehyde-3-phosphate dehydrogenase
RNA-IP: RNA immunoprecipitation
qPCR: quantitative PCR
GFP: green fluorescent protein
NLS: nuclear localization signal
MS2rm: MS2 recognition motif.
